# Differential effects of D-cycloserine and amantadine on motor behavior and D_2/3_ receptor binding in the nigrostriatal and mesolimbic system of the adult rat

**DOI:** 10.1038/s41598-019-52185-7

**Published:** 2019-11-06

**Authors:** Susanne Nikolaus, Hans-Jörg Wittsack, Frithjof Wickrath, Anja Müller-Lutz, Hubertus Hautzel, Markus Beu, Christina Antke, Eduards Mamlins, Maria Angelica De Souza Silva, Joseph P. Huston, Gerald Antoch, Hans-Wilhelm Müller

**Affiliations:** 1 Clinic of Nuclear Medicine, University Hospital Düsseldorf, Heinrich Heine University, Moorenstr. 5, D-40225 Düsseldorf, Germany; 2 Department of Diagnostic and Interventional Radiology, University Hospital Düsseldorf, Heinrich Heine University, Moorenstr. 5, D-40225 Düsseldorf, Germany; 3 Institute for Clinical Diabetology, German Diabetes Center (DDZ), Heinrich-Heine University, Auf´m Hennekamp 65, 40225 Düsseldorf, Germany; 4 Clinic for Nuclear Medicine, University Hospital Essen, Hufelandstraße 55, D-45122 Essen, Germany; 5 Center for Behavioural Neuroscience, Institute of Experimental Psychology, Heinrich-Heine University, Universitätsstr. 1, D-40225 Düsseldorf, Germany

**Keywords:** Dynamical systems, Molecular neuroscience

## Abstract

D-cycloserine (DCS) and amantadine (AMA) act as partial NMDA receptor (R) agonist and antagonist, respectively. In the present study, we compared the effects of DCS and AMA on dopamine D_2/3_R binding in the brain of adult rats in relation to motor behavior. D_2/3_R binding was determined with small animal SPECT in baseline and after challenge with DCS (20 mg/kg) or AMA (40 mg/kg) with [^123^I]IBZM as radioligand. Immediately post-challenge, motor/exploratory behavior was assessed for 30 min in an open field. The regional binding potentials (ratios of the specifically bound compartments to the cerebellar reference region) were computed in baseline and post-challenge. DCS increased D_2/3_R binding in nucleus accumbens, substantia nigra/ventral tegmental area, thalamus, frontal, motor and parietal cortex as well as anterodorsal and posterior hippocampus, whereas AMA decreased D_2/3_R binding in nucleus accumbens, caudateputamen and thalamus. After DCS, ambulation and head-shoulder motility were decreased, while sitting was increased compared to vehicle and AMA. Moreover, DCS increased rearing relative to AMA. The regional elevations of D_2/3_R binding after DCS reflect a reduction of available dopamine throughout the mesolimbocortical system. In contrast, the reductions of D_2/3_R binding after AMA indicate increased dopamine in nucleus accumbens, caudateputamen and thalamus. Findings imply that, after DCS, nigrostriatal and mesolimbic dopamine levels are directly related to motor/exploratory activity, whereas an inverse relationship may be inferred for AMA.

## Introduction

D-cycloserine (DCS; D-4-amino-isoaxazolidinon) and amantadine (AMA; 1-amino-adamantane) act as N-methyl-D-aspartate (NMDA) receptor (R) agonist and antagonist, respectively. DCS binds with high affinity to the glycine_B_ NMDAR subunit^1^ (inhibition constant [K_i_] = 2.33 ± 0.29 μM^2^), and has proven beneficial for the treatment of psychiatric conditions, including schizophrenia^3^, major depressive disorder^4^, anxiety disorder^5,6^ and autism^7^. AMA binds to the phencyclidine NMDAR (K_i_ = 10 μM^8^) and to the opiate σ_1_R (K_i_ = 20 μM^9^). It is mainly applied for the treatment of L-DOPA induced dyskinesia and psychiatric symptoms of Parkinson’s disease^10^, but may also ameliorate major depressive disorder^11^, traumatic brain injury^12^, refractory electrical status epilepticus^13^ and multiple sclerosis fatigue^14^.

In rats, DCS (3 and 15 mg/kg i.p.^15^; 12 mg/kg i.p.^16^; 0.3 or 3 mg/kg i.p.^17^) had no effect on spontaneous locomotor activity^15–17^ or grooming^17^. After higher DCS doses (≥65 mg/kg perorally [p.o.]), however, motor activity reportedly was “slightly depresssed”^18^.

In contrast, AMA (40 or 80 mg/kg i.p.^19^; 50 or 100 mg/kg s.c.^20^; 100 mg/kg i.p.^21^) elevated locomotor activity immediately post-injection with the highest increase after 60 min^19^. A lower dose (20 mg/kg i.p.) was ineffective, whereas a higher dose (160 mg/kg i.p.) induced a depression of motor behavior^19^.

*In vitro*, DCS (10 μM) induced a significant elevation of dopamine (DA) efflux in rat striatal slices^22^, whereas systemic application of DCS (3 mg/kg i.p.) yielded “a slight but non-significant increase” of striatal DA^23^. Moreover, systemic DCS (30 mg/kg i.p.^18^; 300 mg/kg i.p.^24^) augmented γ-amino butyric acid (GABA) in the whole mouse brain, which was related to a decrease of GABA transaminase (GABA-T) levels. DCS (15 mg/kg i.p.^25^; 30 or 100 mg/kg i.p.^18^), furthermore, decreased glutamate (GLU) efflux in the rat amygdala^25^ and in the mouse whole brain^18^. Interestingly, however, no effect was produced in the rat frontal cortex (FC; 50 or 100 mg/kg i.p.^26^).

In rats, systemic AMA (100 mg/kg i.p.) augmented striatal acetylcholine (ACh) and nigral and striatal GABA, starting immediately post-injection^21^. Increases of striatal DA and serotonin (5-HT) concentrations were detectable, but not significant^22^. Also Maj *et al*.^19^ observed no effect on striatal DA after application of lower AMA doses (10 to 80 mg/kg i.p.). Other scientific groups, however, reported a significant augmentation of striatal DA after systemic AMA (40 mg/kg s.c.^27^; 46 or 92 mg/kg i.p.^28^; 100 mg/kg i.p.^29^), also starting immediately post-injection^29^. Likewise, intrastriatal infusion of AMA (0.1 mM or 1 mM) elevated the striatal release of both DA and GLU^29^.

After systemic DCS, so far, no *in vivo* imaging studies of D_2_R-like binding have been performed on either humans or rats. After chronic treatment with AMA (200 mg/day for at least 10 days), two *in vivo* imaging studies of striatal D_2_R-like binding have been conducted on Parkinsonian patients, using [^11^C]raclopride as radioligand^30,31^. Both studies reported a significant elevation of striatal D_2_R-like binding, implying that, at least in Parkinsonian patients, AMA did not increase synaptic DA to an extent sufficient to effect a detecable competition with the exogenous radioligand. Contrarily, in our previous study on healthy rats^32^, AMA challenge with 40 mg/kg i.p. reduced D_2_R-like binding in nucleus accumbens (NAC), caudateputamen (CP) and thalamus (THAL) relative to baseline, while 10 mg/kg diminished D_2_R-like binding in the anterodorsal HIPP (aHIPP).

In the present investigation, we assessed the effects of systemic DCS (20 mg/kg i.p.) on motor/exploratory behaviors and on D_2_R-like binding in regions of the rat nigrostriatal and mesolimbic systems, which are related to motor as well as cognitive and emotional functioning (NAC, CP, THAL, substantia nigra/ventral tegmental area [SN/VTA], FC, motor cortex [MC], parietal cortex [PC], aHIPP, posterior HIPP [pHIPP]) using small animal SPECT and MRI. For all of these areas, including those of HIPP and neocortex, autogradiography studies have confirmed the presence of D_2_R-like binding sites^33–35^. Effects of DCS on D_2_R-like binding were compared to our previous findings on the NMDAR antagonist AMA^32^.

## Materials and Methods

### Animals

Imaging studies of D_2_R-like binding sites were conducted on 38 adult male Wistar rats (ZETT, Heinrich-Heine University, Düsseldorf, Germany), weighing 397 ± 49 g (mean ± standard deviation [SD]; age: 3–4 months). The animals underwent morphological MRI, SPECT measurements in baseline and after injection of DCS or AMA, and behavioral testing after injection of DCS (20 mg/kg: n = 16) or AMA (40 mg/kg: n = 22). MRI and SPECT measurements in baseline and after pharmacological challenges were performed in randomized order and were separated by at least 3 days. Due to seizures or cardial arrest after the administation of the anaesthetic, 5 rats merely underwent behavioral measurements without subsequent D_2_R-like imaging. Behavioral data obtained after DCS and AMA were compared to the behavioral data obtained after vehicle (0.9% saline) in 16 further male rats of the same strain, age (3–4 months) and weight (418 ± 63 g). Behavioral data obtained after 40 mg/kg AMA and saline have been previously published^32,36^.

Rats were kept as previously described (e.g.^32^). The study was performed in accordance with the German Law on the Protection of Animals and the National Institutes of Health guide for the care and use of laboratory animals (NIH Publications No. 8023, revised 1978). The protocol was approved by the regional authority (Landesamt für Natur, Umwelt und Verbraucherschutz, Nordrhein-Westfalen, Recklinghausen, Germany).

### MRI studies

After administration of ketamine hydrochloride (Ketavet®, Pharmacia GmbH, Erlangen, BRD; dose: 50 mg/kg i.p., concentration: 100 mg/ml) and xylazine hydrochloride (Rompun®, Bayer, Leverkusen, BRD; dose: 2.5 mg/kg i.p., concentration: 20 mg/ml), morphological imaging was performed with a dedicated small animal MRI (MRS3000 Pre-clinical MRI, 3.0 T, MR Solutions, Guildford, UK; spatial resolution: 0.25 × 0.25 × 0.69) as previously described^32,37^. Briefly, high-resolution images were obtained with a 3D fast low angle shot (FLASH) sequence^38^.

### Drug treatment

Rats were administered either DCS (Sigma-Aldrich, Taufkirchen, Germany; molecular weight: 102.09 g/mol; dose: 20 mg/kg i.p., concentration: 20 mg/ml; n = 16), AMA hydrochloride (Sigma-Aldrich, Taufkirchen, Germany; molecular weight: 151.25 g/mol; dose: 40 mg/kg i.p., concentration: 40 mg/ml; n = 22) or vehicle (0.9% saline; B. Braun Melsungen AG, Melsungen, Germany; dose: 1 ml/kg i.p.; n = 16).

In former investigations, the doses of 20 mg/kg DCS^39^ and 40 mg/kg AMA^19^ were behaviorally active after systemic administration. Elevations of striatal DA were observed immediately after i.p. injection of DCS^23^ or AMA^29^. Therefore, behavioral measurements were started immediately after administration of either compound.

### SPECT studies

D_2_R-like imaging in baseline and after challenge with DCS or AMA was conducted as previously described^32,36,37,40^. Also the employed small animal SPECT (“TierSPECT”; field of view: 90 mm; sensitivity: 22 for ^123^I; spatial resolution: 3.4 mm for ^123^I) employed in the present study was described in detail elsewhere^41^.

Upon anaesthesia with ketamine hydrochloride (dose: 100 mg/kg i.p., concentration: 100 mg/ml) and xylazine hydrochloride (dose: 5 mg/kg i.p., concentration: 20 mg/ml), [^123^I]S-3-iodo-N-(1-ethyl-2-pyrrolidinyl) methyl-2-hydroxy-6-methoxy benzamide ([^123^I]IBZM; GE Healthcare, München, Germany; activity: 27.9 ± 4.4 MBq, concentration: 3.4 × 10^−9^ g/ml, specific activity: > 74 TBq/mmol at reference time) was injected into the tail vein. This radioligand has a high affinity for binding sites of the D_2_R-like subtype (D_2_R: K_i_ = 1.6 nM, D_3_R: K_i_ = 2.2 nM^42^). Moreover, in investigatons with the “TierSPECT”, we have previously demonstrated its displaceability from the D_2/3_R binding site by endogenous DA (e.g.^36^). In both humans^43^ and rodents^43,44^, under various anaesthetics including ketamine^44^, specific binding of [^123^I]IBZM in the striatum reaches its maximum at about 40 min post-injection and remains stable for up to 2 h. This coincides with the time of maximum striatal DA concentrations after i.p. application of DCS (80 to 160 min post-challenge^23^) and AMA (60 to 90 min post-challenge^29^). In order to account for the respective time courses, data acquisition was started 45 min after radioligand administration (75 min post-challenge) and ended 105 min after radioligand application (135 min post-challenge).

### Behavioral studies

Immediately after administration of DCS, AMA or saline, motor and exploratory behaviors were assessed in an open field (Phenotyper^®^, Noldus Information Technology, Wageningen, The Netherlands; dimensions: 45 × 45 × 56 cm) with EthoVision XT (Noldus Information Technology, Wageningen, The Netherlands) as previously described^32,36,37,40^. Durations (s) and frequencies (n) of ambulation, sitting (as a measure of “passive immobility”^45^), rearing, head-shoulder motility and grooming were rated in blocks of 5 min for a total of 30 min. Subsequent to behavioral tests, rats were anaesthetized as described above and injected [^123^I]IBZM.

### Evaluation of SPECT imaging studies

D_2/3_R imaging data were evaluated with PMOD (version 3.5, PMOD Technologies Ltd., Zürich, Switzerland) as previously described^32,37^. Briefly, for each rat, SPECT and MR images were coregistered. Then, the MR image of each rat was coregistered with the Paxinos standard rat brain MRI^46^ provided by PMOD. The necessary mathematical transformations were used to re-import the SPECT image previously coregistered with the MRI. On the individual overlays of each rat brain SPECT with the Paxinos standard rat brain MRI volumes of interest (VOIs) were defined, as previously described^32,37^. Thereby, the maximum VOI diameters were either in the range of or beyond the spatial resolution of the employed small animal SPECT. Regional BPs were estimated according to the simplified reference tissue model^47^ by computing ratios of radioactivity counts obtained in the specifically-bound compartments (NAC, CP, THAL, SN/VTA, FC, MC, PC, aHIPP and pHIPP) to radioactivity counts in the cerebellar reference VOI.

### Statistical analysis

#### D_2/3_R imaging studies

Distributions of both regional BPs and behavioral data were tested for normality with the non-parametric Kolmogorov-Smirnov test (α ≤ 0.05). Regional BPs were neither uniformly distributed in baseline, nor after DCS or AMA (0.002 ≤ p ≤ 0.200).

Medians and interquartile ranges (25-/75- and 5-/95-percentiles) of regional BPs were computed for both compounds. Moreover, percentual differences of BPs after DCS or AMA relative to baseline were calculated. Regional BPs were compared between baseline and challenge (20 mg/kg DCS or 40 mg/kg mg/kg AMA) with the non-parametric Wilcoxon signed rank test for paired samples (two-tailed, α ≤ 0.05).

#### Behavioral studies

Behavioral variables (duration and frequencies of ambulation, sitting, rearing, head-and-shoulder motility and grooming) were evaluated with two-way analyses of variance (ANOVAs) with the factors “time” (denoting the individual 5-min time bins) and “treatment” (denoting challenge with either 20 mg/kg DCS, 40 mg/kg AMA or saline). In the majority of comparisons over time bins and treatments, the Shapiro-Wilk normality test failed (p < 0.050). Post hoc pairwise comparisons between treatment groups were performed for each variable in the individual time bins with the Holm-Sidak test (overall α ≤ 0.05). Furthermore, Spearman rank correlation coefficients (r; α ≤ 0.05) were calculated for regional radioligand binding and behavioral parameters in the individual time frames (min 1–5, 6–10, 11–15, 16–20, 21–25 and 26–30).

Statistical analysis was performed with IBM SPSS Statistics 23 (IBM SPSS Software Germany, Ehningen, Germany). and SigmaStat (version 3.5, Systat Software Inc., Erkrath, Germany).

## Results

### D_2/3_R binding

**Figure 1:**
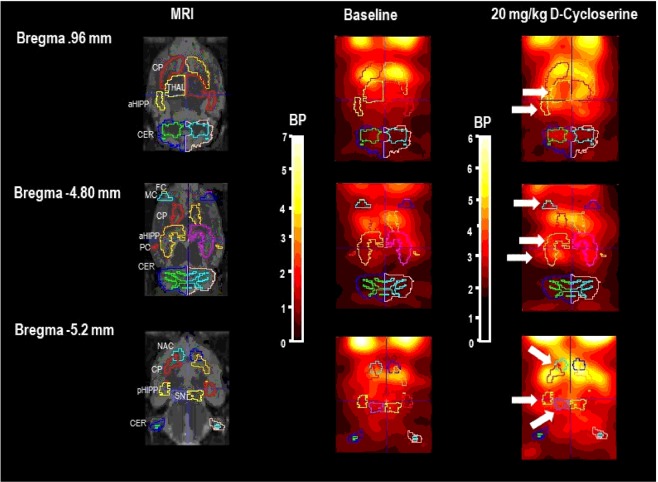
Paxinos standard rat brain MRI and individual D_2/3_R SPECT in baseline and after challenge with the NMDAR agonist **D-cycloserine (20 mg/kg i.p.)** with [^123^I]IBZM as radioligand. *Left columns*: Paxinos standard rat brain MR images (*Schiffer et al., 2006*) at different positions from Bregma together with the standard VOI templates provided by PMOD. *Middle columns*: Series of coronal SPECT slices in a characteristic rat in baseline at the same positions from Bregma. *Right columns:* SPECT slices in the same rat after 20 mg/kg D-cycloserine at the same positions from Bregma. The increases of [^123^I]IBZM accumulation in nucleus accumbens (NAC), substantia nigra/ventral tegmental area (SN), thalamus (THAL), frontal cortex (FC), motor cortex (MC), parietal cortex (PC), anterodorsal hippocampus (aHIPP) and posterior hippocampus (pHIPP) are marked by white arrows. The presented rat also shows an increase of [^123^I]IBZM accumulation in the caudateputamen (CP) compared to baseline, which was not corroborated by the within-group comparison of D_2/3_R binding in baseline and post-challenge. SPECT images show binding potentials (BP). It is understood, that the calculation of BPs is only valid for regions with specific radioligand binding. Image algebra was performed with PMOD (version 3.5, PMOD Technologies Ltd., Zürich, Switzerland).

**Figure 2:**
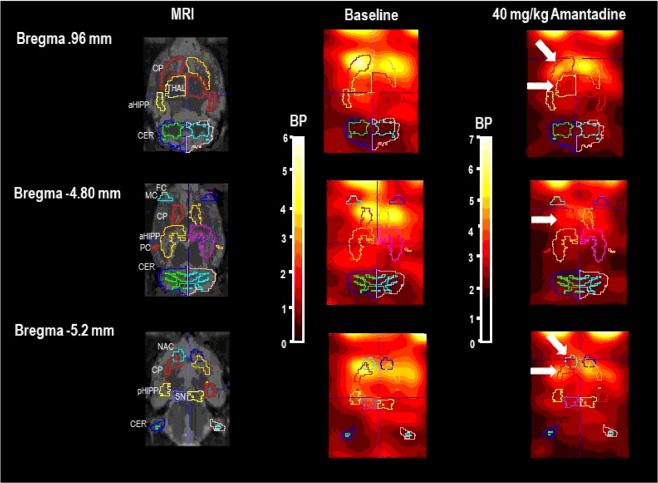
Paxinos standard rat brain MRI and individual D_2/3_R SPECT in baseline and after challenge with the NMDAR antagonist **amantadine (40 mg/kg i.p.)** with [^123^I]IBZM as radioligand. *Left columns*: Paxinos standard rat brain MR images (*Schiffer et al., 2006*) at different positions from Bregma together with the standard VOI templates provided by PMOD. *Middle columns*: Series of coronal SPECT slices in a characteristic rat in baseline at the same positions from Bregma. *Right columns:* SPECT slices in the same rat after 40 mg/kg amantadine at the same positions from Bregma. The reduction of [^123^I]IBZM accumulation in nucleus accumbens (NAC), caudateputamen (CP) and thalamus (THAL) are marked by white arrows. SPECT images show binding potentials (BP). It is understood, that the calculation of BPs is only valid for regions with specific radioligand binding. Image algebra was performed with PMOD (version 3.5, PMOD Technologies Ltd., Zürich, Switzerland). *Further abbreviations* (in alphabetical order): aHIPP), frontal cortex (FC), motor cortex (MC), parietal cortex (PC), posterior hippocampus (pHIPP), substantia nigra/ventral tegmental area (SN).

Figures 1 and 2 show images of the Paxinos standard rat brain MRI atlas^46^ at different positions from Bregma together with the standard VOI templates provided by PMOD (left columns). The next columns show characteristic images of regional [^123^I]IBZM accumulations on coronal slices in baseline (middle) and after challenge (right) with 20 mg/kg DCS (Fig. 1) and 40 mg/kg AMA (Fig. 2), respectively, at the positions from Bregma depicted in the left columns^48^. Baseline and post-challenge scans after both treatments stem from the same rat.

**Figure 3:**
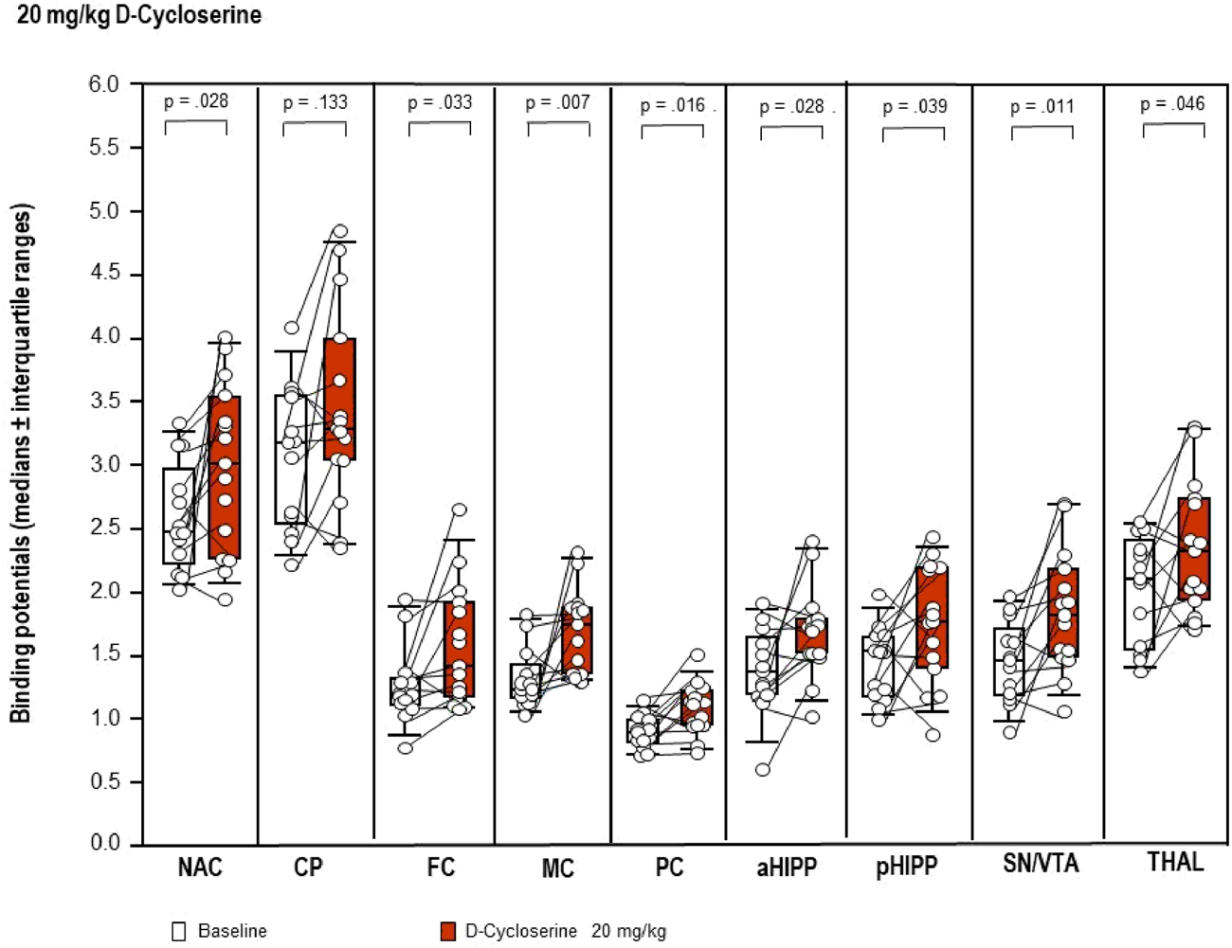
Binding potentials in baseline (white) and after challenge with **20 mg/kg D-cycloserine** (red). Rendered are medians and 25-/75- (boxes) and 9-/95-quartiles (whiskers). The circles represent the individual animals. For the paired comparisons, the respective p values are given (Wilcoxon signed rank test for paired samples, two-tailed, α = 0.05). *Abbreviations* (in alphabetical order): aHIPP, anterodorsal hippocampus; FC, frontal cortex; MC, motor cortex; PC, parietal cortex; pHIPP, posterior hippocampus; SN/VTA, substantia nigra/ventral tegmental area.

After 20 mg/kg DCS (Fig. 3), BPs were significantly augmented in NAC (+22%, p = 0.028), SN/VTA (+24%, p = 0.011), THAL (+10%, p = 0.046), FC (+19%, p = 0.033), MC (+41%, p = 0.007), PC (+25%, p = 0.016), aHIPP (+25%, p = 0.028) and pHIPP (+16%, p = 0.039) relative to baseline. No significant alteration was observed in the CP (p = 0.133).

**Figure 4:**
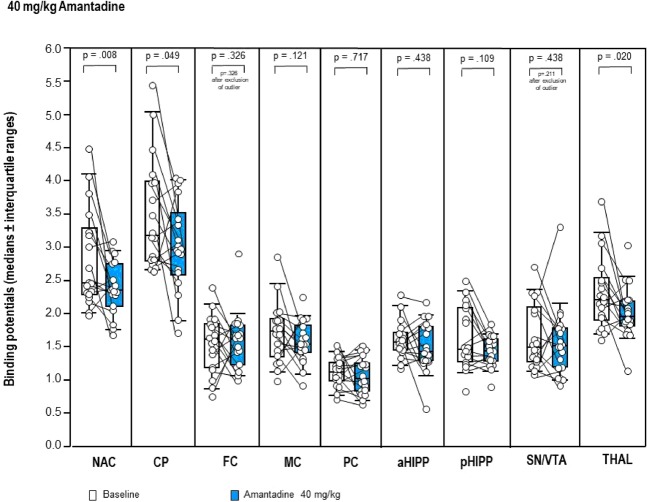
Binding potentials in baseline (white) and after challenge with **40 mg/kg amantadine** (blue). Rendered are medians and 25-/75- (boxes) and 9-/95-quartiles (whiskers). The circles represent the individual animals. For the paired comparisons, the respective p values are given (Wilcoxon signed rank test for paired samples, two-tailed, α = 0.05). *Abbreviations* (in alphabetical order): aHIPP, anterodorsal hippocampus; CP, caudateputamen; FC, frontal cortex; MC, motor cortex; NAC, nucleus accumbens; PC, parietal cortex; pHIPP, posterior hippocampus; SN/VTA, substantia nigra/ventral tegmental area; THAL, thalamus.

In contrast, the NMDAR antagonistic AMA (Fig. 4) induced significant reductions of the BP in NAC (−5%, p = 0.008), CP (−7%, p = 0.049) and THAL (−12%, p = 0.020) compared to baseline. No differences between 40 mg/kg AMA and baseline were observed in SN/VTA, FC, MC, PC, aHIPP and pHIPP (0.109 ≤ p ≤ 0.438).

### Motor and exploratory behaviors

For ambulation duration, the two-way ANOVAs yielded significant effects of “treatment” (p < 0.001), “time” (p < 0.001) and “treatment x time” (p = 0.005), whereas, for ambulation frequency, only effects of “time” (p < 0.001) and “treatment x time” (p = 0.001) were found. Both sitting duration and frequency yielded significant effects of “treatment” (p < 0.001, each), “time” (p < 0.001, each) and “treatment x time” (p < 0.001 and p = 0.014, respectively). This also held for rearing duration and frequency (“treatment”: p < 0.001, each; “time”: p < 0.001, each; “treatment x time”: p < 0.001, each), and for both duration and frequency of head-shoulder motility (“treatment”: p < 0.001, each; “time”: p = 0.041 and p < 0.001, respectively; “treatment x time”: p < 0.001, each). The analysis of grooming duration yielded significant effects of “treatment” (p < 0.001) and “time” (p < 0.001), whereas, for grooming frequency, significant effects of “time” (p = 0.002) and “treatment x time” (p = 0.013) were obtained.

**Figure 5:**
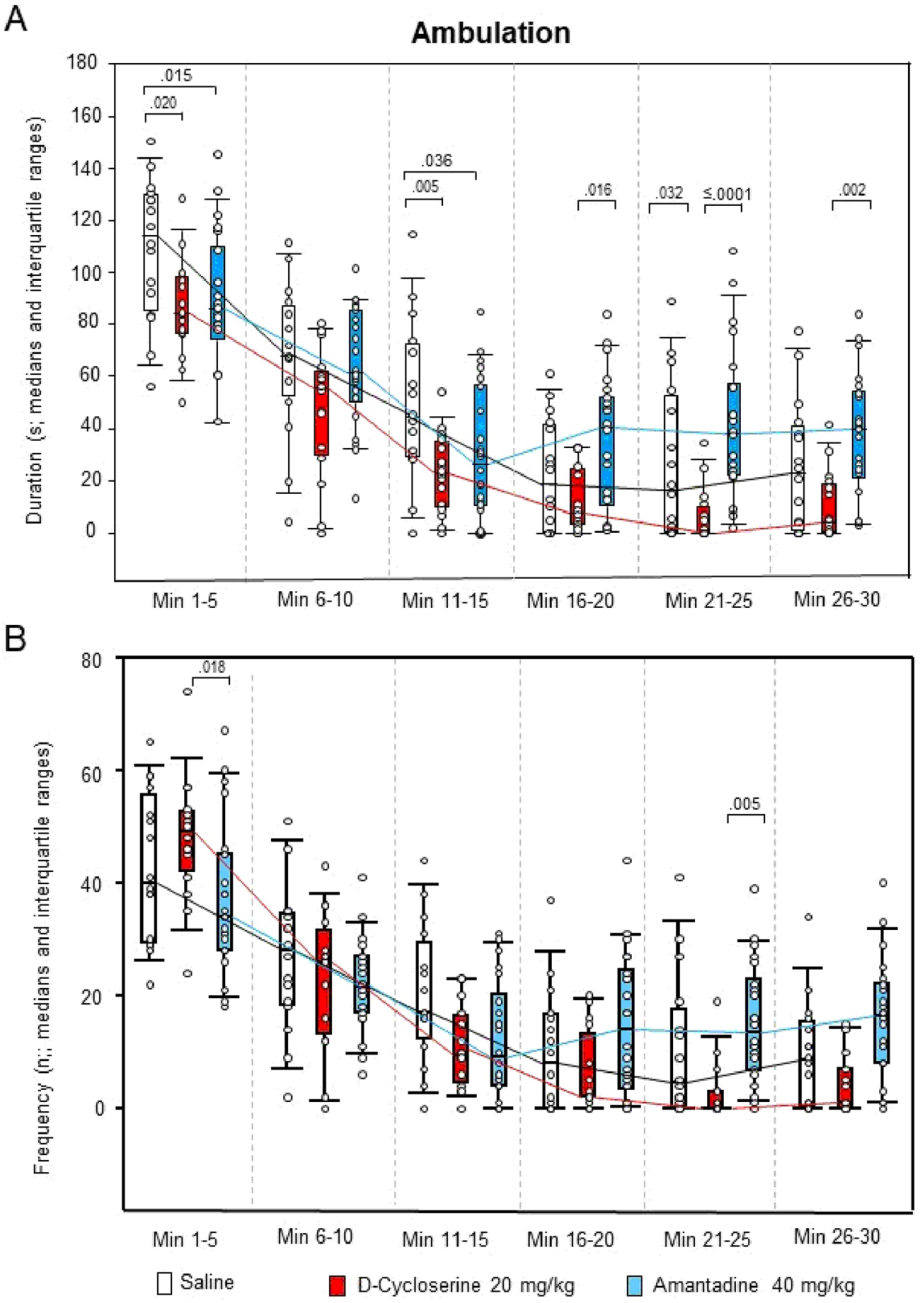
Ambulation. Duration (s) and frequency (n) after vehicle (0.9% saline; white), 10 mg/10 D-cycloserine (red) and 40 mg/kg amantadine (blue). The figure shows box and whisker plots of median ambulation durations (**A**) and frequencies (**B**) in the individual 5-min time bins. 25-/75-percentiles are given in the boxes, while 5-/95-percentiles are represented by the whiskers. The circles represent the individual animals. The medians in each time bin are connected by black (saline), red (D-cycloserine) and blue lines (amantadine). Between-group differences were assessed using the Holm-Sidak test (two-tailed). Significant p values are given.

**Figure 6:**
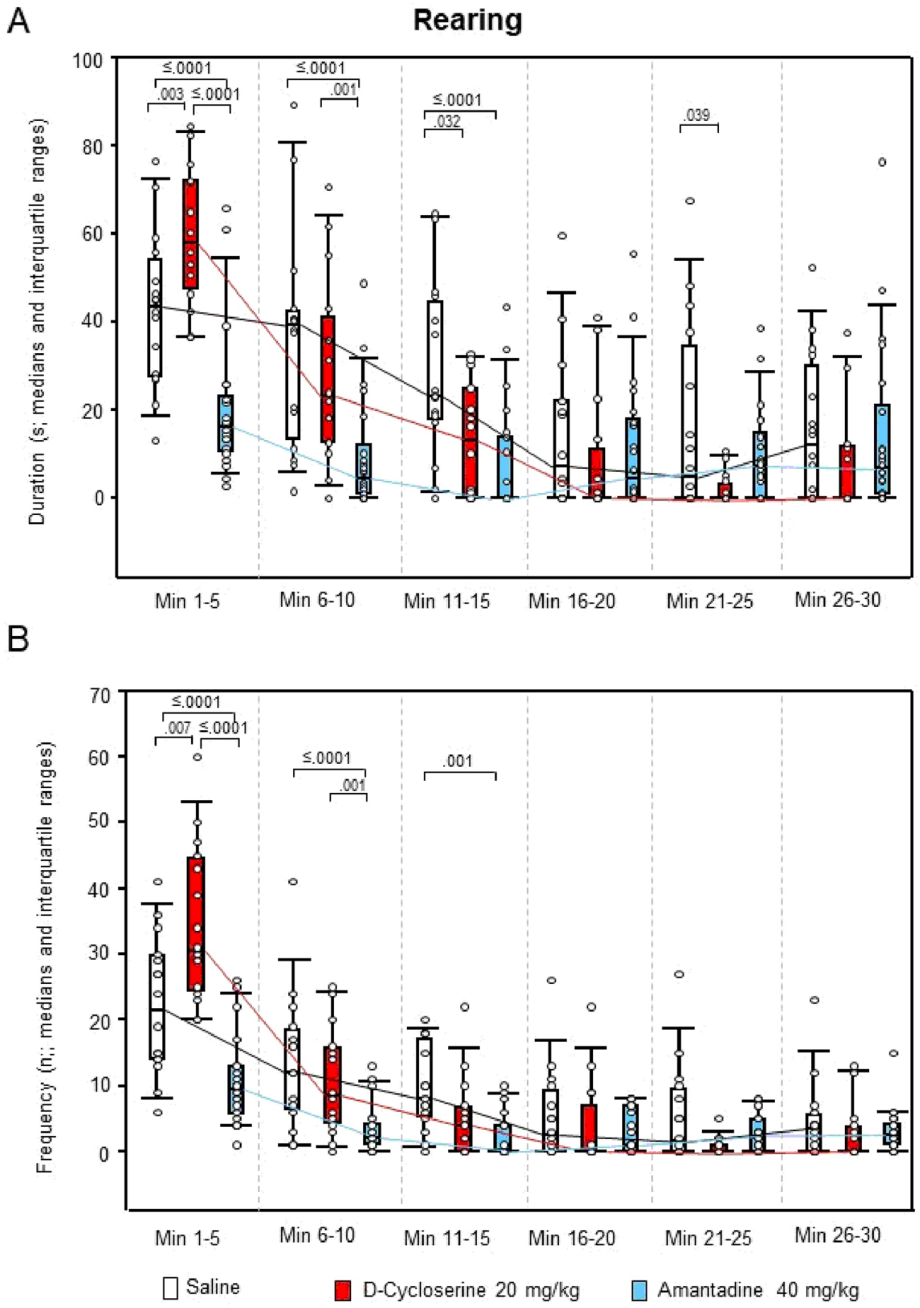
Rearing. Duration (s) and Frequency (n) after vehicle (0.9% saline, white), 10 mg/10 D-cycloserine (red) and 40 mg/kg amantadine (blue). The figure shows box and whisker plots of median rearing durations (**A**) and frequencies (**B**) in the individual 5-min time bins. 25-/75-percentiles are given in the boxes, while 5-/95-percentiles are represented by the whiskers. The circles represent the individual animals. The medians in each time bin are connected by black (saline), red (D-cycloserine) and blue lines (amantadine). Between-group differences were assessed using the Holm-Sidak test (two-tailed). Significant p values are given.

**Figure 7:**
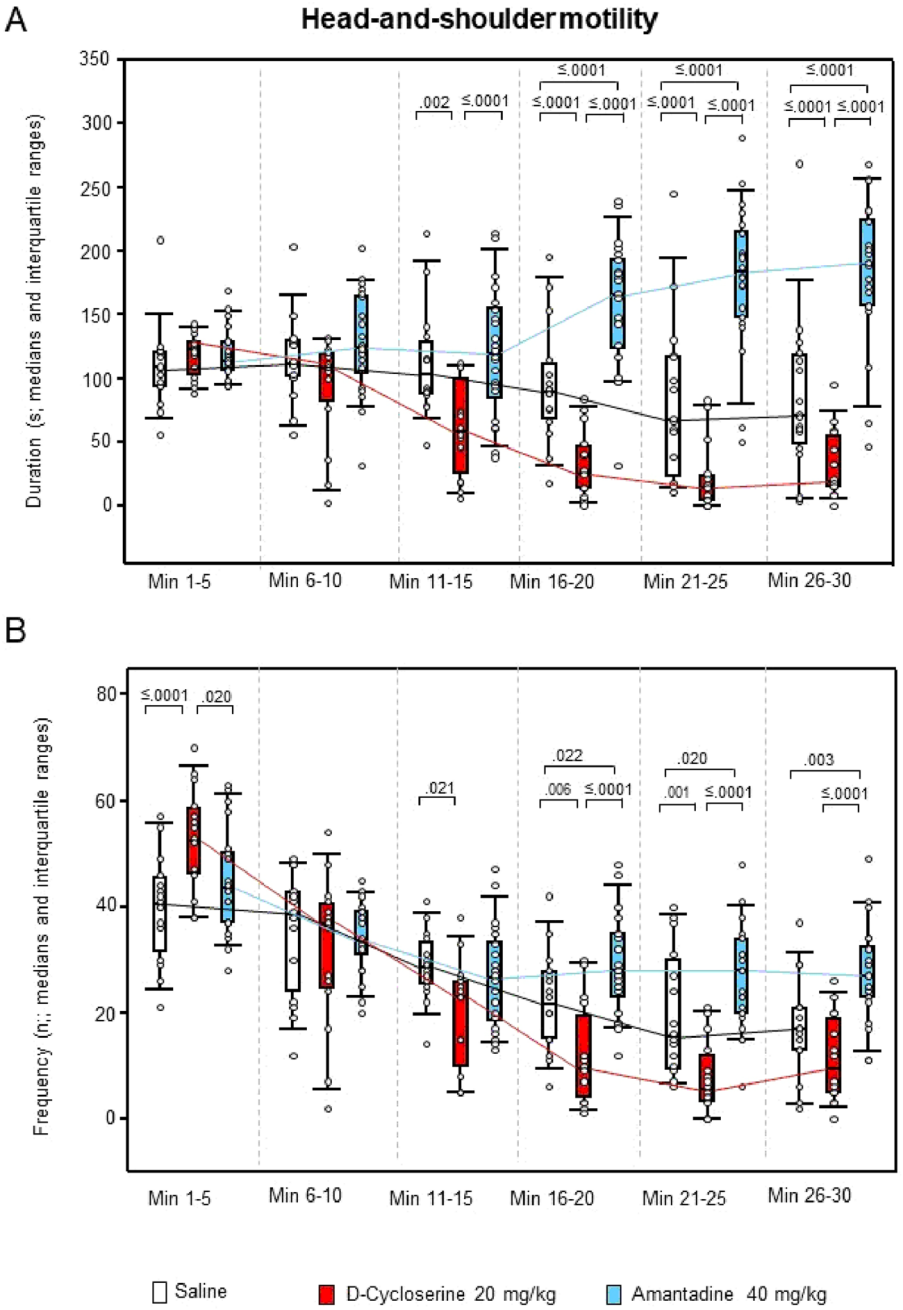
Head-shoulder motility. Duration (s) and Frequency (n) after vehicle (0.9% saline, white), 10 mg/10 D-cycloserine (red) and 40 mg/kg amantadine (blue). The figure shows box and whisker plots of the median durations (**A**) and frequencies (**B**) of head-shoulder motility during in the individual 5-min time bins. 25-/75-percentiles are given in the boxes, while 5-/95-percentiles are represented by the whiskers. The circles represent the individual animals. The medians in each time bin are connected by black (saline), red (D-cycloserine) and blue lines (amantadine). Between-group differences were assessed using the Holm-Sidak test (two-tailed). Significant p values are given.

**Figure 8:**
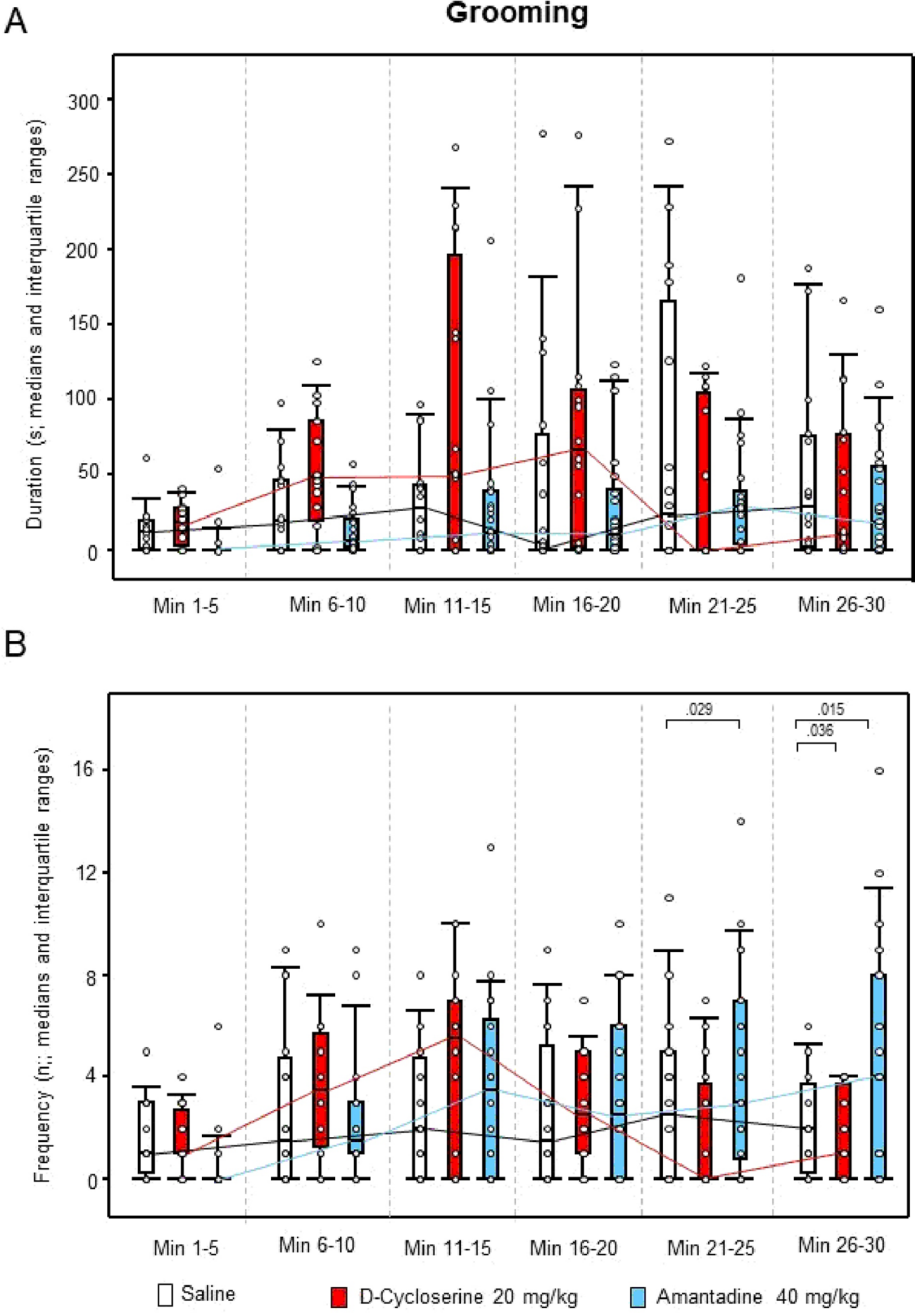
Grooming. Duration (s) and Frequency (n) after vehicle (0.9% saline, white), 10 mg/10 D-cycloserine (red) and 40 mg/kg amantadine (blue). The figure shows box and whisker plots of the median durations (**A**) and frequencies (**B**) of grooming in the individual 5-min time bins. 25-/75-percentiles are given in the boxes, while 5-/95-percentiles are represented by the whiskers. The circles represent the individual animals. The medians in each time bin are connected by black (saline), red (D-cycloserine) and blue lines (amantadine). Between-group differences were assessed using the Holm-Sidak test (two-tailed). Significant p values are given.

**Figure 9:**
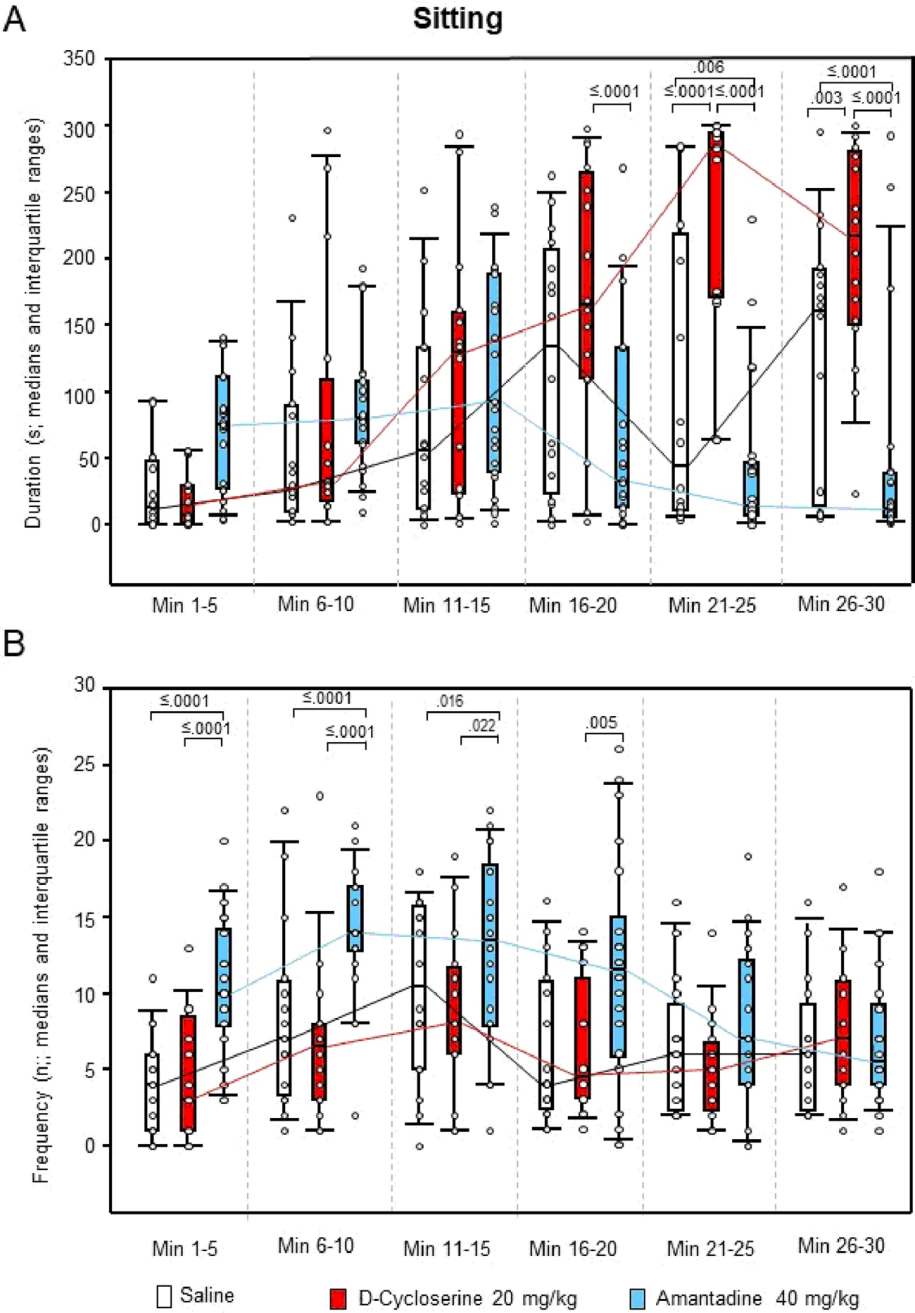
Sitting. Duration (s) and frequency (n) after vehicle (0.9% saline; white), 10 mg/10 D-cycloserine (red) and 40 mg/kg amantadine (blue). The figure shows box and whisker plots of median sitting durations (**A**) and frequencies (**B**) in the individual 5-min time bins. 25-/75-percentiles are given in the boxes, while 5-/95-percentiles are represented by the whiskers. The circles represent the individual animals. The medians in each time bin are connected by black (saline), red (D-cycloserine) and blue lines (amantadine). Between-group differences were assessed using the Holm-Sidak test (two-tailed). Significant p values are given.

After DCS, ambulation duration (min 1–5, 11–15 and 21–25; Fig. 5), rearing duration (min 11–15 and 21–25; Fig. 6), both duration (min 11–30) and frequency (min 1–5 and 11–25) of head-shoulder motility (Fig. 7) and grooming frequency (min 26–30; Fig. 8) were decreased compared to saline, while sitting duration (min 21–39; Fig. 9) and both rearing duration and frequency in min 1–5 (Fig. 6) were augmented.

Correlation analysis revealed the following associations between behavioral parameters and regional D_2/3_R after 20 mg/kg DCS: lower sitting duration in min 1–5 ↔ higher D_2/3_R in MC (r = −0.517); lower sitting frequency in min 16–20 ↔ lower D_2/3_R in CP (r = 0.525), FC (r = 0.673) and MC (r = 0.567); lower rearing frequency in min 6–10 ↔ higher D_2/3_R in SN/VTA (r = −0.581); lower frequency of head-shoulder motility in min 1–5 ↔ higher D_2/3_R in NAC (r = −0.624), CP (r = −0.687), THAL (r = −0.536), SN/VTA (r = −0.551), FC (−0.504) and pHIPP (−0.608); lower grooming duration in min 6–10 ↔ lower D_2/3_R in MC (r = 0.590); lower grooming duration in min 26–30 ↔ higher D_2/3_R in NAC (r = −0.517) and lower grooming frequency in min 1–5 and 11–15 ↔ lower D_2/3_R in PC (r = 0.560) and pHIPP (r = 0.532), respectively.

After AMA, sitting frequency was increased from min 1–15, while sitting duration was decreased from min 21–30 (Fig. 9). Furthermore, duration and frequency (min 26–30, each; Fig. 7) of head-shoulder motility were elevated relative to saline, whereas ambulation duration (min 1–5 and 11–15; Fig. 5) and both rearing duration and frequency (min 1–15, each; Fig. 6) were reduced.

After 40 mg/kg AMA, the following associations between behavior and D_2/3_R were obtained: lower ambulation duration in min 1–5 and 11–15 ↔ lower D_2/3_R in SN/VTA (r = 0.561) and pHIPP (r = 0.603), respectively; lower ambulation frequency in min 1–5 ↔ lower D_2/3_R in NAC (r = 0.596), CP (r = 0.513), THAL (r = 0.498) and SN/VTA (r = 464); lower sitting duration in min 1–5 and 26–30 ↔ higher D_2/3_R in SN/VTA (r = −0.504) and pHIPP (r = −0.468), respectively; lower sitting frequency in min 6–10 ↔ higher D_2/3_R in NAC (r = 0.−606) and CP (r = −0.464); lower rearing duration in min 1–5 ↔ lower D_2/3_R in NAC (r = 0.521); lower rearing duration in min 21–25 ↔ higher D_2/3_R in THAL (r = −0.525); lower rearing frequency in min 21–25 ↔ higher D_2/3_R in THAL (r = −0.490); lower frequency of head-shoulder motility in min 1–5 and 11–15 ↔ lower D_2/3_R in NAC (r = 0.502), CP (r = 0.509), THAL (r = 0.598) and pHIPP (r = 0.604), respectively; lower grooming frequency in min 1–5, 6–10 and 11–15 ↔ lower D_2/3_R in NAC (r = 0.475 and 0.500), CP (r = 0.569) and PC (r = 0.550), respectively.

Comparison between NMDAR agonistic and antagonistic treatment yielded reduced ambulation duration (min 16–30; Fig. 5), sitting frequency (min 1–20; Fig. 9), duration of head-shoulder motilty (min 11–30; Fig. 7) and grooming frequency (min 21–30; Fig. 8) after DCS relative to AMA, while sitting duration was increased (min 16–30; Fig. 9). Moreover, after DCS, ambulation frequency was initially (min 1–5; Fig. 5) augmented compared to AMA, but declined in min 21–25. Similarly, the frequency of head-shoulder motility was initially (min 1–5; Fig. 7) elevated, but reduced from min 16–30.

### Ethical approval

All applicable international, national, and/or institutional guidelines for the care and use of animals were followed. This article does not contain any studies with human participants performed by any of the authors.

## Discussion

Challenge with the NMDAR agonist DCS in a dose of 20 mg/kg significantly increased D_2/3_R binding in NAC (+22%), SN/VTA (+24%), THAL (+10%), FC (+19%), MC (+41%), PC (+25%), aHIPP (+25%) and pHIPP (+16%), whereas challenge with the NMDAR antagonist AMA in a dose of 40 mg/kg reduced D_2/3_R binding in NAC (−5%), CP (−7%) and THAL (−12%).

In precedent studies on rats, systemic treatment with the DA precursor L-DOPA^36^, the DA reuptake inhibitor methylphenidate^49^, and the GABA_A_R agonist muscimol^37,40^ diminished [^123^]IBZM binding to the rat D_2/3_R. The augmentation of DA concentrations in the synaptic cleft is common to all of these compounds. Since [^123^]IBZM competes with endogenous DA molecules for D_2/3_R binding sites, the observed decreases of D_2/3_R binding may be conceived to reflect increased levels of synaptic DA^50^. Therefore, it may be surmised that, also in the present study, the AMA-induced regional reductions of D_2/3_R binding were due to elevated DA concentrations in these areas, whereas the observed regional increases of D_2/3_R binding after pre-treatment with DCS indicate reductions of available DA.

This is the first study, which assessed the effects of DCS on subcortical and neocortical DA in rats with a non-invasive *in vivo* imaging approach. Until now, the effect of DCS challenge on DA has only been studied in the rat CP, where either no effect^23^ or a significant elevation of DA efflux^22^ was observed. The latter is in contrast with our findings, which did not show an alteration of D_2/3_R binding in the CP after 20 mg/kg DCS. Likely reasons for this inconsistency are the differences in methods: firstly, we performed *in vivo* SPECT, while Bennett and Gronier^22^ assessed striatal homogenates with high pressure liquid chromatography; secondly, we administered DCS systemically, while Bennett and Gronier^22^ incubated striatal slices; and, thirdly, we used adult rats with a mean weight of 397 ± 49 g, while Bennett and Gronier^22^ employed adolesent animals, weighing between 250 and 350 g.

Also the effect of AMA on DA has only been studied in the rat CP. As detailed before^32^, our finding of elevated DA in the CP following administration of AMA is consistent with the results of Scatton *et al*.^27^, Quack *et al*.^28^ and Takahashi *et al*.^29^ also obtained after systemic AMA (40 and 100 mg/kg). It disagrees, however, with the findings of Maj *et al*.^19^ and Bak *et al*.^21^, who failed to detect alterations of striatal DA after 10 to 100 mg/kg. In all of these studies, either immature (110–150 g^19^) or adolescent (250–300 g^21,27–29^ rats were used, precluding age as relevant factor for the discrepancy of outcomes. All of these studies employed either invasive *in vivo* methods such as microdialysis^28,29^ or *ex vivo* methods such as spectrofluorometry^19,21^ and ion exchange chromatography of striatal preparations^27^. Scatton *et al*.^27^, however, sacrificed their rats 2 h post-challenge, whereas in the studies of Bak *et al*.^21^ and Maj *et al*.^19^ animals were killed only 1 h after systemic AMA. This indicates that a time of 1 h post-challenge may not be sufficient to induce detectable changes in neostriatal DA levels, at least if AMA action in living compartments is excluded by the chosen *ex vivo* approach.

Effects of the NMDAR agonistic DCS and the NMDAR antagonistic AMA on motor/exploratory parameters can be summarized as follows: (1) ambulation duration was decreased after both DCS (min 1–5, 11–15 and 21–25 and AMA (min 1–5 and 11–15), while ambulation frequency was unaltered after both treatments; (2) sitting duration was increased after DCS (min 21–30), but decreased after AMA (min 21–30), while sitting frequency was unaltered after DCS, but increased after AMA (min 1–15); (3) both rearing duration and frequency were initially increased after DCS (min 1–5, each) but decreased after AMA (min 1–15, each); (4) both duration (min 11–30) and frequency of head-shoulder motility (min 1–5 and 16–25) were decreased after DCS, but increased after AMA (min 16–30, each); and, finally, (5) grooming duration was unaltered after DCS and AMA, while grooming frequency was decreased after DCS (min 26–30) and unaltered after AMA. Moreover, comparisons between DCS and AMA revealed (1) decreased ambulation duration (min 16–30); (2) increased sitting duration (min 16–30), but decreased sitting frequency (min 1–20); (3) increased rearing duration and frequency (min 1–10, both); (4) decreased duration (min 11–30) and frequency of head-shoulder motility (min 16–30) and, finally, (5) decreased grooming frequency (min 21–30) after DCS relative to AMA. Thereby, interestingly, the frequencies of both ambulation and head-shoulder motility were elevated during the first 5 min post-injection relative to AMA.

The present result of significantly decreased ambulation after challenge with DCS compared to saline contradicts previous studies, which reported either no effect or merely a slight depression of spontaneous locomotor activity after systemic treatment with 0.3 to 65 mg/kg DCS^15–18^. Also the finding of decreased ambulation after challenge with AMA does not agree with previous fndings, showing an elevation of locomotor activity after systemic treatment with 40 to 100 mg/kg AMA^19–21^. Only the present finding of unaffected grooming duration confirms previous results obtained after 0.3 and 3 mg/kg DCS^17^.

Pharmacological effects on motor function in rodents are strongly dependent on age: the DA precursor L-DOPA, for instance, increased motor activity in neonatal (5 to 8 days old^51^) and immature rats (18 to 20 days old^52^) after doses of 12.5 to 50 mg/kg and 150 mg/kg, respectively, whereas motor activity was diminished in adult animals (25 to 30 days of age^52^). In the present study, rats were considerably older (approximately 4 months old and weighing 397 ± 49 g) compared to the other investigations on DCS and AMA, in which adolescent (250–300 g^15^; 250 ± 50 g^16^; 200–250 g^17^; 200 g^18^; 110–115 g^19^; 100–120 g^20^; average weight of 250 g^21^) animals were used. As a consequence, the present discrepancies may be accounted for by the difference of ages between samples in conjunction with NMDAR agonistic and antagonistic action on synaptic DA levels. The effects of DCS and AMA on both regional D_2/3_R binding and motor/exploratory parameters must be assessed in future investigations in rats of different ages in order to shed further light on this matter.

It is not surprising that the NMDAR agonist DCS and the NMDAR antagonist AMA exert opposite actions on D_2/3_R binding. Striking, however, are the differences in regional contributions: while AMA affects merely NAC, CP and THAL, DCS acts on the site of origin of DA fibers (SN/VTA) as well as on target regions of DAergic projections throughout the mesolimbic and nigrostriatal system (NAC, THAL, neocortex, HIPP) with the exception of the CP.

In rodents, DCS increased GABA efflux in the mouse whole brain^18,24^. Moreover, decreases of GLU levels were observed in the rat amygdala^25^ as well as in the mouse whole brain^18^, whereas no effect was detected in the rat FC^26^. This implies that overall alterations of GABAergic and GLUergic input to the nigrostriatal and mesolimbic target regions of ascending and descending fibers incurred a net decline of DA, reflected by the observed increases of D_2_R binding in NAC, SN/VTA, THAL, neocortex and HIPP.

As far as can be inferred from precedent investigations, the major difference between DCS and AMA action on cortical and subcortical DA levels is that GLU appears to be either unaffected or decreased by the former^18,25,26^ but increased by the latter^29^. As previously outlined in more detail^32^, it may be assumed that AMA (contrarily to DCS) increases GLUergic input to the target regions of corticostriatal and corticomesolimbic projections, thus augmenting DA efflux in CP, NAC and THAL. It may be hypothesized that these differences in GLUergic and DAergic activation are related to the observed behavioral differences, namely decreased rearing duration and frequency (min 1–10, each) and increased sitting frequency (min 1–20) after AMA compared to DCS. Moreover, also in the second half of the testing time, after AMA, exploration was primarily performed by the sitting animal merely moving its head and shoulders (min 11–30).

In the direct pathway (CP - pars reticulata of the SN/internal globus pallidus) DA disinhibits GABAergic neurons, incurring an activiation of the mesencephalic, diencephalic and brainstem motor centers, whereas, in the indirect pathway (CP - external globus pallidus/subthalamic nucleus - pars recticulata of the SN/internal globus pallidus), GABAergic neurons are inhibited by DA, resulting in a suppression of motor activity^53^. Moreover, the NAC with its afferents to the limbic system and its efferents to the GP acts as a limbic-motor interface, which is pivotal for the translation of emotional and motivational states into action^54^. Correlation analysis revealed that, after DCS, high sitting frequency (at 15 min post-challenge) predicted high D_2/3_R binding (and low DA) in CP, FC and MC (at 75 min post-challenge). Moreover, a low frequency of head-shoulder motility (immediately post-challenge) predicted high D_2/3_R binding (and low DA) in NAC, CP, THAL, SN/VTA, FC and pHIPP (at 75 min post-challenge). Contrarily, after AMA, a high sitting frequency (at 6 min post-challenge) predicted low D_2/3_R binding in NAC and CP (at 75 min post-challenge), whereas low durations and/or frequencies of ambulation and head-shoulder motility (immediately post-challenge) predicted low D_2/3_R binding in NAC, CP, THAL and SN/VTA (at 75 min post-challenge). This infers that after DCS and AMA, the altered levels of DA in the individual regions of the nigrostriatal and mesolimbic pathway within the first 15 min post-injection differentially affected motor neurons in conjunction with emotional/motivational states. Thereby, after DCS, lower regional DA concentrations produced the effect that rats were less able (and/or less “motivated”) to ambulate or explore their environment by head-shoulder movements, but more able (and/or more “motivated”) to exhibit rearing behavior. In contrast, after AMA, higher regional DA levels induced a general behavioral depression characterized by decreased rearing and increased sitting, while, consistently, exploration mainly consisted of head-and-shoulder movements. The question still remains to be solved, however, in as much the regional BPs (and DA levels) at the time of *in vivo* imaging correspond to the DA levels at the time of data acquisition in the open field. Future investigations are needed, in which behavior are assessed for a longer time than 30 min post-challenge. Moreover, regional D_2/3_R binding should be determined in different sets of animals at various times after [^123^I]IBZM application.

In the present study, findings may have been biased by the employment of the NMDAR antagonist ketamine as anaesthetic. Since ketamine has previously been shown to enhance DA release in rats (e.g.^55–57^), it can not be excluded that DA release elicited by ketamine actually reduced the amounts of visible regional D_2/3_ receptor binding after both DCS and AMA. Effects on neostriatal and/or ventrostriatal DA, however, are exerted by practically all known anaesthetics, including pentobarbital, propofol, halothane, chloral hydrate and isoflurane (for review see^58^). Therefore, we decided to maintain the usage of ketamine, which was employed in all our previous investigations. Since also this possible pitfall concerns the outcome of SPECT measurements both in baseline and post-challenge, the obtained BPs remain comparable between conditions.

## Conclusion

Taken together, in adult rats, DCS increased D_2/3_R binding in NAC, SN/VTA, THAL, FC, MC, PC, aHIPP and pHIPP, whereas AMA decreased D_2/3_R binding in NAC, CP and THAL. The elevations of D_2/3_R binding after DCS reflect a reduction of available DA throughout the nigrostriatal and mesolimbic system, while the reductions of D_2/3_R binding after AMA indicate an increased availability of DA in NAC, CP and THAL. Findings imply a direct relationship between nigrostriatal and mesolimbic DA levels and motor/exploratory activity after DCS, but an inverse relationship after AMA.
